# SEU Hardened D Flip-Flop Design with Low Area Overhead

**DOI:** 10.3390/mi14101836

**Published:** 2023-09-27

**Authors:** Chenyu Yin, Yulun Zhou, Hongxia Liu, Qi Xiang

**Affiliations:** Key Laboratory for Wide Band Gap Semiconductor Materials and Devices of Education, School of Microelectronics, Xidian University, Xi’an 710071, China; yin_chenyu@163.com (C.Y.); ylzhou_xd@163.com (Y.Z.); q77xiang@163.com (Q.X.)

**Keywords:** D flip-flop (DFF), single event upset (SEU), reliability, hardening, fully depleted silicon-on-insulator (FDSOI)

## Abstract

D flip-flop (DFF) is the basic unit of sequential logic in digital circuits. However, because of an internal cross-coupled inverter pair, it can easily appear as a single event upset (SEU) when hit by high-energy particles, resulting in the error in the value stored in the flip-flop. On this basis, a new structure D flip-flop is proposed in this paper. This flip-flop uses an asymmetric scheme in which the master–slave latch adopts different hardening structures. By sacrificing circuit speed in exchange for stronger SEU fortification capability, the SEU threshold of this structure is improved by 10 times compared to traditional D flip-flops. It has also been compared with Dual Interlocked Storage Elements (DICEs), and it saves the area cost of six transistors compared to the DICE structure. Under the same operating conditions, the average power consumption and peak power consumption are, respectively, 9.8% and 18.8% lower than those of the DICE circuit, making it suitable for soft radiation environments where high circuit speed is not a critical requirement.

## 1. Introduction

Digital integrated circuits are widely used today, and D flip-flop (DFF) is the basic unit of sequential logic in digital circuits. However, because there is a cross-coupled inverter [1] similar to SRAM inside the flip-flop, if the sensitive node collects a certain charge when it is hit by particles, it will cause the internal stored value to flip [2]. In addition, with the development of manufacturing process technology, the feature size and operating voltage of the device are gradually reduced, and the probability of single event upset (SEU) in the device is also increasing [3]. The occurrence of SEU means that the value stored in the flip-flop is wrong at this time. If the error is read by the system, it may cause the system to crash. Therefore, in order to improve the reliability of the circuit, we need to protect against possible SEU.

Triple modular redundancy (TMR) is a commonly used protection method [4,5,6], which makes three copies of sensitive units in the circuit. When one of the units fails, the voter can select the correct value. TMR has a good protection effect against SEU, but it will bring a large area overhead. Error-correcting codes (ECC) are another commonly used SEU hardening method, which is widely used in today’s processors [7,8,9]. The ECC hardening method involves writing the check codes generated by the encoder at the same time as writing data to the storage unit, reading the data and the corresponding check code together when reading, and decoding them to detect and correct the error of the data error. However, the error correction capability of ECC coding is limited, and if a large number of errors occur, it cannot be corrected.

To prevent a large number of errors, it can be considered to harden the basic unit of the flip-flop. The common hardening schemes can be divided into two categories: adding redundant units and introducing negative feedback mechanisms [10,11]. The DICE structure is proposed based on these two hardening methods [12], which has strong resistance against SEU. However, it also incurs a significant area overhead in the circuit. Furthermore, most of the traditional hardening schemes use the symmetrical structure of master–slave latches; this paper studies the basic structure of conventional D flip-flop and finds that the SEU hardening capability of the master latch in the flip-flop of this structure is stronger than that of the slave latch. On this basis, this paper introduces a novel structure of D flip-flop with the following characteristics:

1. This DFF utilizes an asymmetric scheme. Different hardening structures are employed for the master and slave latches based on their SEU resistance capabilities. The circuit requires six fewer transistors compared to the DICE structure, effectively reducing the circuit area overhead. Furthermore, the average power consumption and peak power consumption of the circuit are 9.8% and 18.8% lower, respectively, compared to a DICE DFF under identical operating conditions.
2. The structure exhibits strong SEU hardening capabilities. Compared to traditional D-flip-flops, this structure achieves an almost ten-fold increase in the SEU flip threshold. It provides the same hardening effect as DICE under single particle injection with Linear Energy Transfer (LET) ranging from 0 to 0.7 pc/μm.

## 2. Conventional D Flip-Flop

### 2.1. Working Principle

**Figure 1:**
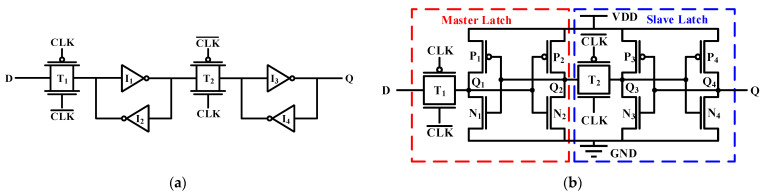
Scheme of the conventional D flip-flop [13]: (**a**) gate-level (**b**) transistor-level.

There are many structures of D flip-flops, which can be implemented by NAND gates, transmission gates and other structures. In this study, the D flip-flop implemented by transmission gates shown in Figure 1 is used as the basic structure for research. This structure does not introduce transmission gates between the inverters, which saves the number of transistors and reduces the clock load but requires adjusting the size of the transistors to ensure that new values can be written. The transistor-level circuit of a conventional D flip-flop is shown in Figure 1b. A unit contains 12 transistors, which are divided into a master latch and slave latch.

The working principle of the D flip-flop is as follows: when the clock signal is low, the transmission gate T_1_ is turned on, and the two internal cross-coupled inverters I_1_ and I_2_ can receive and store the value input by the D node. When the clock signal becomes high, the transmission gate T_2_ is turned on, and the two cross-coupled inverters I_3_ and I_4_ can receive and store the value passed in by the transmission gate T_2_ and transmit it to the Q node at the same time. It is equivalent to the D flip-flop sampling from the D node and spreading to the Q node on the rising clock edge.

### 2.2. Simulation of Basic Function

In this study, HSPICE (Version R-2020.12) is used to simulate the basic function of the D flip-flop. Subsequent simulations were performed using the 22 nm fully depleted silicon-on-insulator (FDSOI) device model from Global Foundries. We obtained the single event transient (SET) current at different LET through FDSOI device simulations. Using the Weibull function model, we fitted the current source model with MATLAB and finally implemented the single-particle circuit simulation by adding the current source model to the circuit. Table 1 presents the detailed parameters of the DFF circuit. By driving the clock and D signal, the D flip-flop is tested twice for sampling logic 1 and sampling logic 0, respectively.

**Figure 2:**
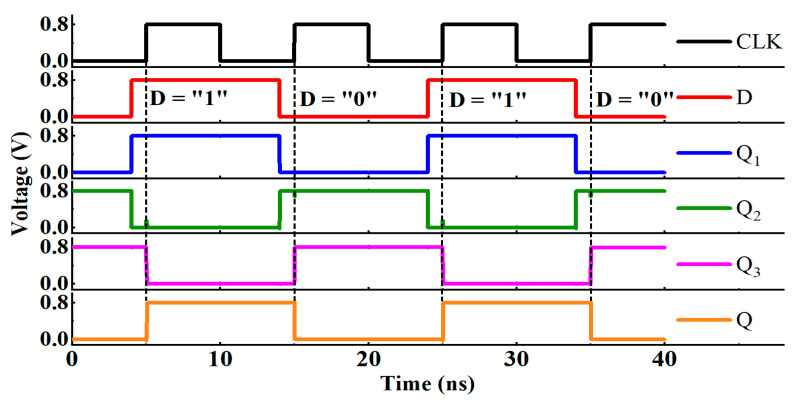
Timing diagram of the conventional D flip-flop.

As shown in Figure 2, when the clock is low, the voltages of Q_1_ and Q_2_ nodes follow the change of the D node, the master latch successfully stores the value of the D node, and when the clock is high, the slave latch successfully receives the master latch value. The value of the D node sampled at the rising clock edge is finally successfully transmitted to the Q node, which is consistent with expectations.

**Figure 3:**
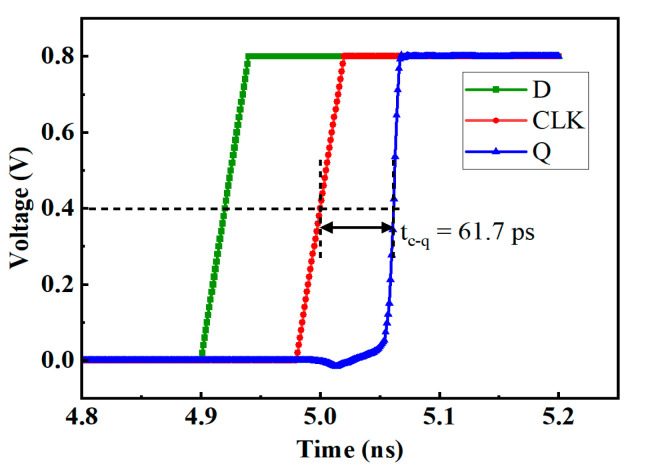
Propagation delay of the conventional D flip-flop.

The propagation delay of the D flip-flop is defined as the time difference between the moment when the clock signal voltage rises to 50% VDD and the moment when the Q node voltage rises to 50% VDD. As shown in Figure 3, the propagation delay of a conventional D flip-flop is 61.7 ps.

### 2.3. SEU Simulation

This section will simulate the SEU of the master latch and the slave latch of the D flip-flop, respectively. Firstly, the high-energy particle incident simulation is carried out on the 22 nm device model. The current waveform of a single tube at each LET is obtained by simulation. Subsequently, the SET current was fitted using the Weibull function, resulting in a current source model that conforms to a Weibull distribution. Each LET corresponds to a set of Weibull model parameters. Finally, DFF circuit-level SEU simulation is realized by adding a Weibull current source to DFF circuit. By changing the parameters, the current source generates different currents, and the correlation between the current source model and LET is realized.

When the clock is low, because the transmission gate T_1_ is turned on, if the voltage of the Q_1_ node changes, it will quickly return to the voltage of the D node. In addition, the change in the voltage of the storage node of the master latch will not affect the Q node because the transmission gate T_2_ is turned off.

**Figure 4:**
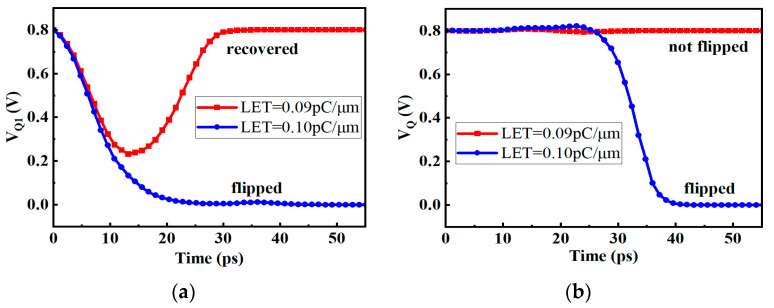
The voltage diagram of pulse current injected into Q_1_ node (**a**) Q_1_ node (**b**) Q node.

However, when the clock is high, the transmission gate T_1_ will be turned off. If the pulse current is injected into the Q_1_ node at this time, as shown in Figure 4a, the voltage of the Q_1_ is prone to flipping, and the flipping linear energy transfer (LET) threshold is between 0.09 and 0.10 pC/μm. As shown in Figure 4b, because the transmission gate T_2_ is on, the inversion of the Q_1_ node will affect the Q node, causing the output of the D flip-flop to also flip. At this time, if the wrong value is read, it may cause system errors.

**Figure 5:**
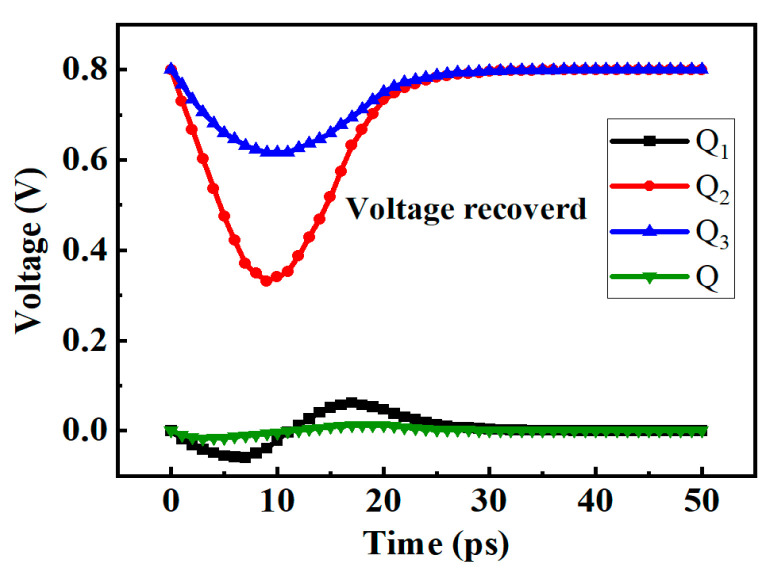
The voltage diagram of pulse current injected into Q_2_ node.

Different from the Q_1_ node, when the clock is high, if a high-energy pulse current with an LET value of 0.7 pC/μm is injected into the Q_2_ node, the result is shown in Figure 5, and the voltage of the Q_2_ has not yet been reversed. The reason is that the transmission gate T_2_ is on, the pulse current of the Q_2_ node has been reduced when it is transmitted to the Q_3_ node through the transmission gate, and the change in the voltage of the Q_3_ node is not enough to cause the slave latch to flip, so the voltage of the Q_2_ node will gradually recover when the pulse current disappears.

From the above results, it can be concluded that when the master latch of the D flip-flop is affected by SEU, if the clock signal is low, it will not affect the output of the D flip-flop, and if the clock is high, only Q_1_ is a sensitive node. For the slave latch, when the clock is low, the transmission gate T_2_ is turned off, and the voltage of the internal node will not be affected by the master latch. However, when the pulse current is directly injected into the slave latch, the voltage will be flipped. The LET flipping threshold of the Q_3_ node is between 0.07 and 0.08 pC/μm, and that of the Q node is between 0.06 and 0.07 pC/μm.

**Figure 6:**
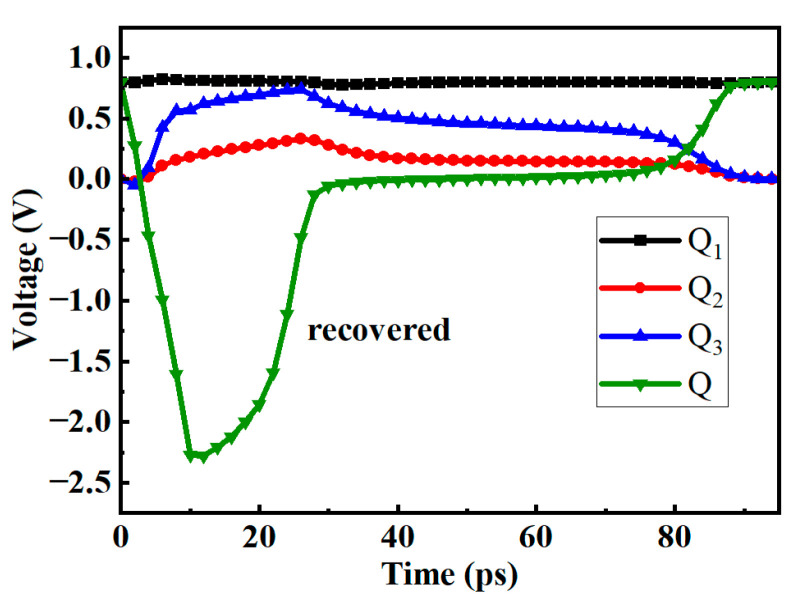
The voltage diagram of pulse current injected into Q node.

When the clock is high, a high-energy pulse current is injected into the Q node; as shown in Figure 6, the Q node voltage drops rapidly and the Q_3_ node voltage rises, but after passing through the transmission gate, the voltage change of the Q_2_ node decreases, and it does not cause reverse in the master latch. Then, after the pulse current disappears, the voltages of each node begin to recover, but the recovery time becomes longer than other cases, about 100 ps. If the pulse current is injected into the Q_3_ node, there is a similar phenomenon, and the recovery principle is the same.

Summarizing the above results, it can be concluded that the slave latch of the D flip-flop is easier to appear as SEU in the low state of the clock, and the flipping threshold is lower than that of the master latch in the high state of the clock. The worst-case flipping threshold of the D flip-flop is between 0.06 and 0.07 pC/μm, which is significantly improved compared to 6T-SRAM (about 0.01 pC/μm). Table 2 summarizes the SEU hardening capability of a conventional D flip-flop. For the master latch of the flip-flop, the change of the voltage of the master latch when the clock is low will not affect the Q node as the output. When the clock is high, only the Q_1_ node becomes the SEU’s sensitive node.

For the slave latch, the two nodes of the cross-coupled inverter are sensitive nodes when the clock is low, and the flipping threshold is lower than that of the master latch; that is, SEU is more likely to occur. Therefore, it can be concluded that the SEU hardening capability of the slave latch is weaker than that of the master latch in the conventional D flip-flop.

## 3. Proposed D Flip-Flop

### 3.1. Asymmetric Reinforcement Circuit Structure

**Figure 7:**
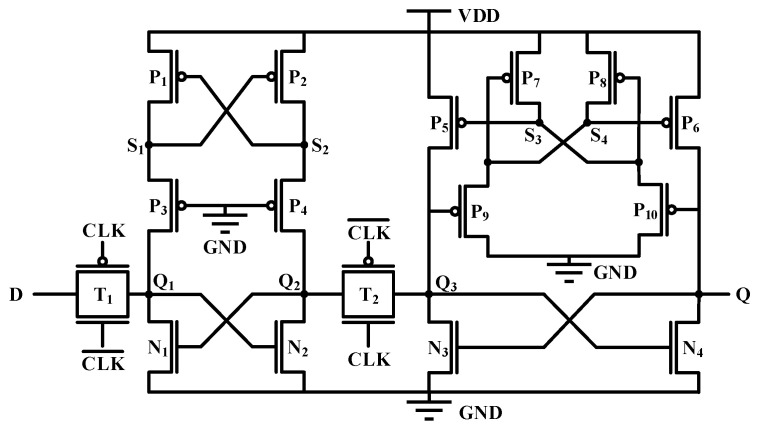
Scheme of proposed D flip-flop.

Based on the research findings from the previous section, it can be concluded that for hardening D flip-flops, a structure with a better hardening effect than the master latch can be adopted for the slave latch. Moreover, compared with 6T-SRAM, the flip threshold of the conventional D flip-flop itself is higher. Based on the above two points, this study proposes a new structure D flip-flop, as shown in Figure 7. Table 3 presents the detailed parameters of the asymmetric reinforcement circuit. For the main latch with strong SEU immunity, a redundancy fortification method is adopted. Two normally-on PMOS transistors are added to the Q_1_ and Q_2_ nodes of the circuit to mitigate the impact of SET current. For the slave latch with weaker SEU immunity, we added four additional PMOS transistors to the original circuit. When the Q point voltage is high and the Q_3_ point voltage is low, the P_9_ transistor conducts, causing P_7_ to conduct as well, making the S_4_ node low and the S_3_ node high. When the Q point or the Q_3_ point is subjected to an SET current, the influence on the S_3_ and S_4_ nodes is relatively small. If the logic of the S_3_ and S_4_ can remain unchanged, the voltage at Q and Q_3_ points can be restored to their initial states by affecting P_5_ and P_6_, thereby maintaining the logic integrity of the circuit. The area overhead of the structure is not large and it has better SEU hardening ability.

The results of simulation show that the propagation delay of this structure is 461.5 ps, which is larger than that of the conventional D flip-flop. The reason is that the slave latch contains P_9_ and P_10_ transistors with the drain grounded, which causes the voltage change of the node in the slave latch to be relatively slow, which is the shortcoming of this structure.

### 3.2. SEU Simulation

**Figure 8:**
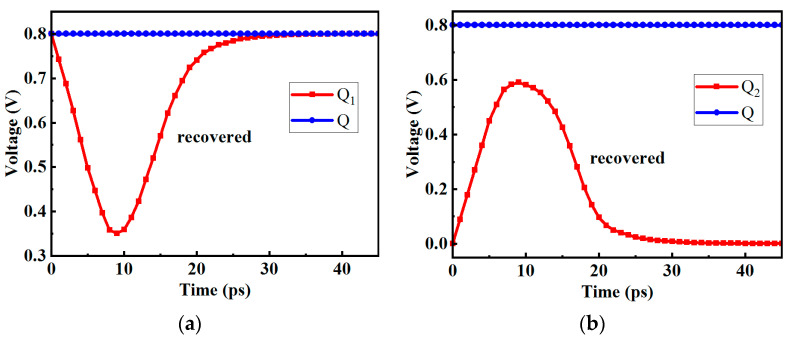
The voltage diagram of the new structure D flip-flop when the pulse current is injected into (**a**) Q_1_ (**b**) Q_2_.

When the clock is low, pulse currents are injected into the Q_1_ and Q_2_ nodes, respectively, and the results are shown in Figure 8. The voltages of the Q_1_ and Q_2_ will recover quickly, because the transmission gate T_1_ is turned on. At the same time, the voltage change in the master latch will not affect the Q node because the transmission gate T_2_ is turned off, so the output of flip-flop will not be affected.

In this structure, two additional P transistors are introduced to the master latch. Assume that the initial logic of the Q_1_ node is 1, and Q_2_ is 0. If the N_1_ transistor is hit by particles, the logic of the Q_1_ node changes from 1 to 0, but because there will be a voltage difference between the source and drain of the P_3_, the voltage of the S_1_ node will not change rapidly to 0, so that the P_2_ will not be turned on immediately and the Q_2_ node will also flip, achieving the SEU hardening effect.

**Figure 9:**
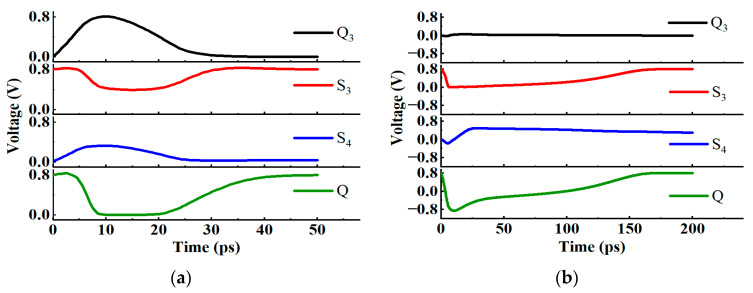

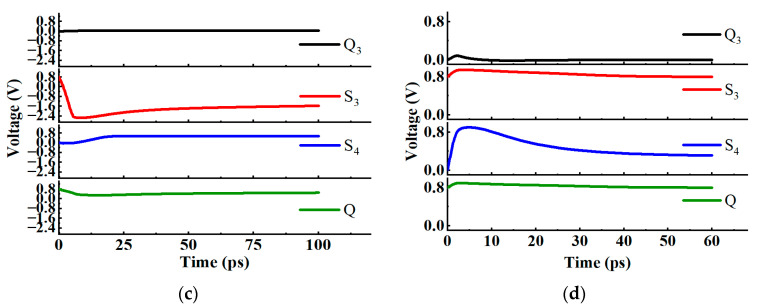
The voltage diagram of the new structure D flip-flop when injected with an SET current of LET = 0.7 pc/μm at (**a**) Q_3_ (**b**) Q (**c**) S_3_ (**d**) S_4_.

Figure 9 shows the voltage variations at different nodes of the circuit when subjected to an SET current with LET = 0.7 pc/μm. Assuming that the potential of the Q_3_ node rises from logic 0 to 1, the N_4_ is turned on, the Q node voltage drops, and the P_10_ is turned on. However, because the P_10_ is connected to the ground, and there is a voltage difference of the transistor itself, at this time, the voltage of the S_3_ node will not drop to 0, so that the P_5_ will not be turned on. After the pulse current disappears, the voltage of the Q_3_ node will return to its original value. In an ideal hardening scheme, the width of the P_7_ and P_8_ should be greater than that of the P_9_ and P_10_ so that when the voltage of the Q_3_ or Q node changes, the voltage of the S_3_ and S_4_ nodes hardly changes. But this will affect the normal writing function of the D flip-flop, so the P_7_ and P_8_ should not be too wide.

The results demonstrate that the circuit exhibits strong resistance to SEU. When subjected to an SET current with LET = 0.7 pc/μm, the voltages at various nodes of the circuit can still recover to their initial state within 30~170 ps, indicating that the circuit has an LET flip threshold greater than 0.7 pc/μm. In contrast, the LET flip threshold of a regular DFF circuit is typically between 0.06 and 0.07 pc/μm. The fortified circuit shows a tenfold increase in the flip threshold compared to the standard DFF circuit.

### 3.3. Circuit Comparison

**Figure 10:**
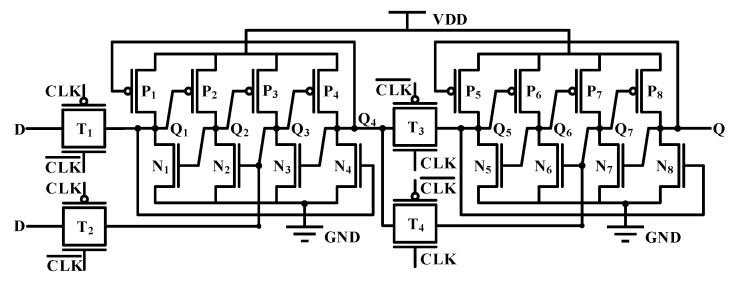
Scheme of DICE structure D flip-flop.

The DICE circuit, renowned for its exceptional radiation tolerance, has found extensive applications in reinforced circuits [14]. We uniformly employed a 22 nm FDSOI process library to construct the circuit models. The DFF of the DICE structure is shown in Figure 10, in which the cross-coupled inverter inside the flip-flop is replaced by the DICE structure. At the same time, due to the isolation structure of DICE, two additional transmission gates need to be added to realize data transmission. The DICE structure adopts the internal isolation method to realize the reinforcement of the SEU. The D flip-flop of this structure contains 24 transistors in total, 12 transistors are added compared with the conventional D flip-flop, and the area overhead is relatively large. A comprehensive set of circuit parameters is presented in Table 4 for reference.

**Figure 11:**
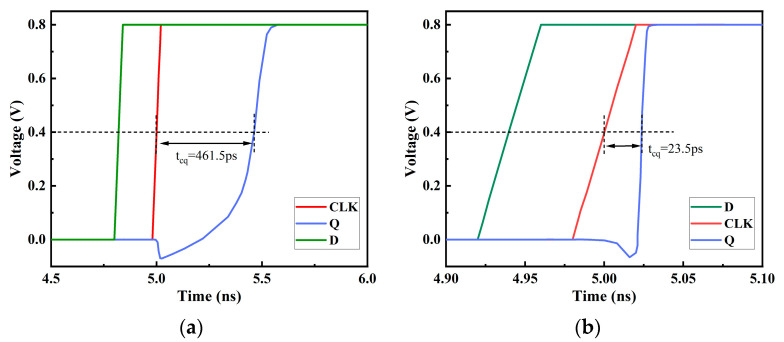
Propagation delay of the (**a**) proposed circuit and (**b**) DICE D flip-flop.

Figure 11 presents a comparison of the propagation delays of the two circuits. The results demonstrate that the DICE structure’s D flip-flop exhibits a very low propagation delay, measuring only 23.5 ps. Compared to the traditional D flip-flop, it represents a reduction of approximately 62%, indicating an improved performance. This improvement primarily stems from the inclusion of two transmission gates in each latch, which accelerates the storage speed of the latch voltage. The asymmetrically hardened DFF exhibits a propagation delay of 461.5 ps, which is approximately 7.5 times that of the traditional D flip-flop. This suggests that the circuit is not suitable for high-speed applications.

**Figure 12:**
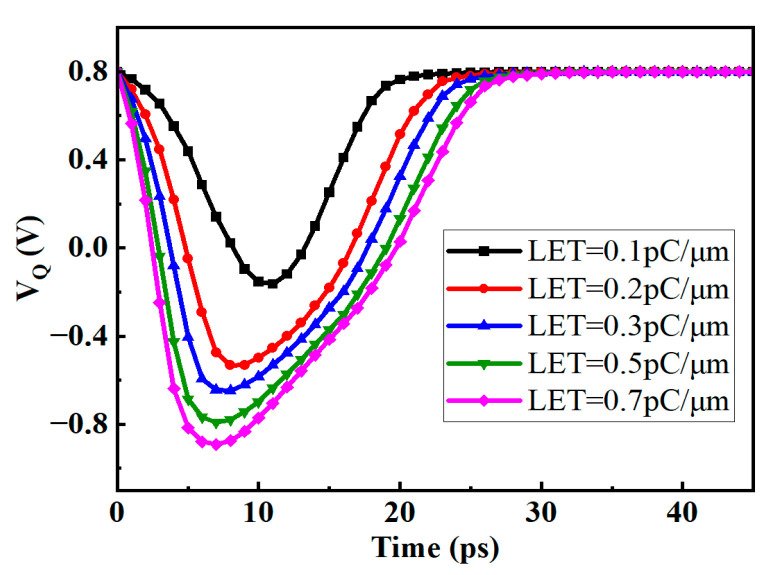
The voltage diagram of pulse current injected into Q node of DICE structure D flip-flop.

**Figure 13:**
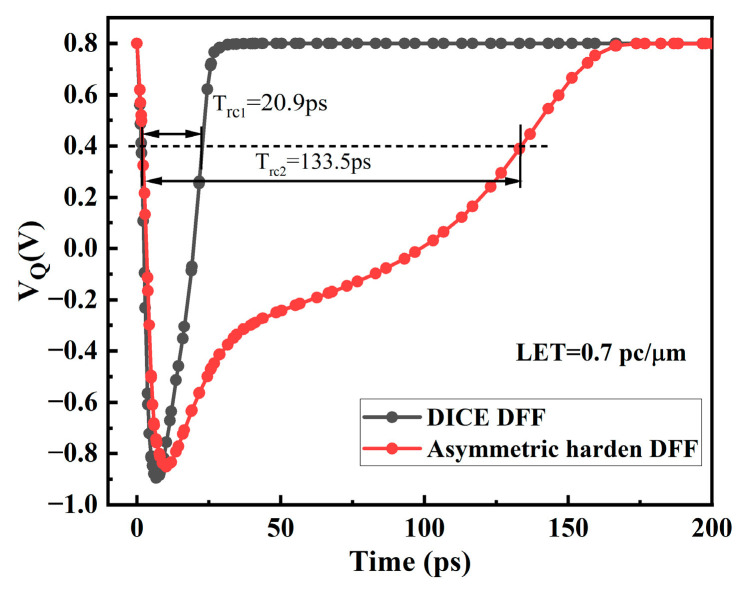
The voltage variations at the Q nodes of DICE and the proposed circuit were observed when an SET current with LET = 0.7 pc/μm was injected.

The DICE structure D flip-flop is similar to the SRAM of this structure, and each node can resist SEU. As shown in Figure 12, under various energy pulse current injections, the voltage of the Q node can eventually return to the original value. The DICE structure D flip-flop has a smaller propagation delay and better SEU hardening ability, but it will bring a larger area overhead. Figure 13 illustrates the voltage variations at the Q nodes of the two circuits under SET current with LET = 0.7 pc/μm (particle energy loss) conditions. It can be observed that both the asymmetrically fortified DFF and DICE DFF can maintain circuit logic integrity under high-energy particle impacts. In this figure, we define the recovery time (T_rc_) as the time difference between the voltage dropping to 50% VDD and recovering to 50% VDD. According to the results, it is found that DICE exhibits a significantly shorter recovery time than the asymmetrically fortified circuit, only requiring approximately 20.9 ps, while the asymmetrically fortified circuit needs 133.5 ps. Nevertheless, both circuits demonstrate strong capabilities in recovering from soft errors.

**Figure 14:**
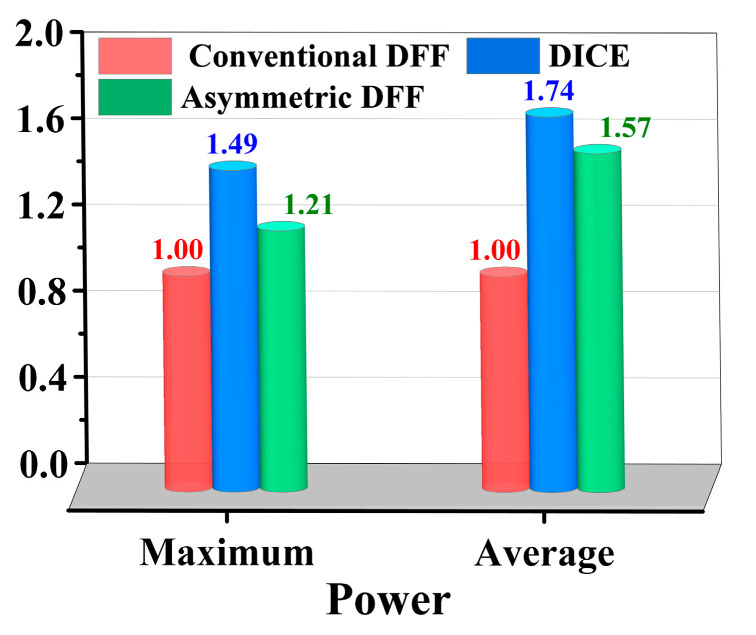
Power consumption comparison of different D flip-flop structures.

The power consumption of the two circuits is also compared. The average power and peak power of these three circuits within the first 40 ps under the same operating conditions were calculated using Hspice software. Under the same operating conditions, the power consumption of the conventional DFF is defined as 1. Figure 14 shows the power consumption comparison of the three circuits. Due to the advantages of the asymmetrically fortified structure, the circuit has six fewer transistors compared to DICE, resulting in lower average and peak power consumption. Under the same operating conditions, the average power and peak power of the asymmetrically fortified DFF are 1.57 and 1.21, respectively, while those of DICE are 1.74 and 1.49. Therefore, it can be concluded that the asymmetrically fortified circuit has a significant advantage in power control. The complete comparative results are listed in Table 5.

Table 5 shows the various results of D flip-flops with different structures. Compared with conventional D flip-flops, the proposed structure D flip-flop has increased propagation delay, but the SEU hardening ability has been enhanced. In addition, referring to experimental data [15] from the 65 nm FDSOI DICE DFF, the soft error rate of the DICE circuit is significantly lower compared to the conventional DFF and TMR circuits. Experimental data [16,17] from the 22 nm FDSOI DICE indicate that the SEU cross-section of DICE is an order of magnitude lower than that of the traditional DFF in static state. The asymmetrically fortified DFF exhibits a similar performance to DICE in SEU simulations, indirectly demonstrating the reliability of this circuit.

## 4. Conclusions

Because of the cross-coupled inverters in the D flip-flop, the sensitive node is easy to appear as an SEU when it is hit by particles, resulting in errors in its internal storage values. In this study, it is found that the SEU hardening ability of the master latch in the D flip-flop of this structure is stronger than that of the slave latch. Therefore, for the hardening of the D flip-flop, a structure in which the hardening effect of the slave latch is better than that of the master latch can be used. Moreover, compared with 6T-SRAM, the flip threshold of the conventional D flip-flop itself is higher. If the DICE structure D flip-flop is used, it will bring excess SEU hardening capability, and the overall area of the circuit will be larger. Based on the above two points, a new structure D flip-flop is proposed in this paper. The flip-flop uses an asymmetric scheme in which master–slave latches adopt different hardening structures. This structure has strong SEU hardening ability. Compared to the DICE structure, it achieves a reduction in area occupancy by six transistors, which is accompanied by lower power consumption. However, the propagation delay is 461.5 ps with some loss in performance. The D flip-flop of this structure is suitable for application scenarios that do not require high performance but have a limited area. The simulation results demonstrate that our proposed circuit can withstand SET current shocks at LET = 0.7 pc/μm. However, it is possible that in practical application scenarios, such a high level of SEU fortification may not be necessary, and the circuit’s SEU resistance performance may exceed the requirements. Therefore, further consideration will be given to improving the transistor sizes in the circuit to achieve an enhancement in circuit speed.

## Figures and Tables

**Table 1:** Transistor parameter information for the conventional DFF circuit.

Device	W (nm)	L (nm)	W/L
N_1_, N_3_, N_4_	92	22	4.2
N_2_, N_5_~N_7_, P_1_, P_3_, P_4_	184		8.4
P_2_, P_5_~P_7_	368		16.8

**Table 2:** SEU hardening capability of conventional D flip-flop.

Injection Position	Clock Signal	SEU Hardening Ability (Whether the Q Node Is Flipped)
Master latch	Low	Can resist SEU
	High	Q_1_ is sensitive node
Slave latch	Low	Both Q_3_ and Q are sensitive nodes
	High	Can resist SEU

**Table 3:** Transistor parameter information for the proposed DFF circuit.

Device	W (nm)	L (nm)	W/L
N_1_, N_4_	92	22	4.2
N_13_	184		8.4
N_2_, N_11_, N_12_	276		12.6
P_1_, P_5_~P_8_, N_3_	46		2.1
P_3_, P_9_, P_10_, P_13_	368		16.8
P_2_, P_4_, P_11_, P_12_	552		25.1

**Table 4:** Transistor parameter information for the DICE DFF circuit.

Device	W (nm)	L (nm)	W/L
N_1_~N_3_, N_5_~N_8_, N_13_	100	22	4.6
N_9_~N_12_	300		13.7
P_5_	50		2.3
N_4_, P_1_~P_3_, P_6_~P_8_, P_13_	200		9.1
P_4_	400		18.2
P_9_~P_12_	600		27.3

**Table 5:** Comparison of the results of D flip-flop with different structures.

Structures	Number of Transistors	Propagation Delay (ps)	SEU Threshold (pc/um)	Average Power	Maximum Power
Conventional	12	61.7	0.06~0.07	1	1
DICE	24	23.5	>0.7	1.74	1.49
Proposed	18	461.5	>0.7	1.57	1.21

## Data Availability

Not applicable.
